# A Protective Effect of Pirfenidone in Lung Fibroblast–Endothelial Cell Network via Inhibition of Rho-Kinase Activity

**DOI:** 10.3390/biomedicines11082259

**Published:** 2023-08-12

**Authors:** Yusuke Nakamura, Yasuo Shimizu, Mio Fujimaki-Shiraishi, Nobuhiko Uchida, Akihiro Takemasa, Seiji Niho

**Affiliations:** Department of Pulmonary Medicine and Clinical Immunology, School of Medicine, Dokkyo Medical University, 880 Kitakobayashi, Mibu 321-0293, Tochigi, Japan; nakamuyu@dokkyomed.ac.jp (Y.N.); fujikasen3089@gmail.com (M.F.-S.); n-uchida@dokkyomed.ac.jp (N.U.); takemasa@dokkyomed.ac.jp (A.T.); siniho@dokkyomed.ac.jp (S.N.)

**Keywords:** interstitial pneumonia, angiogenesis, pirfenidone, transforming growth factor β (TGF-β), Rho-kinase, ROCK, pulmonary fibrosis

## Abstract

Pulmonary fibrosis is a life-threatening disease that has been attributed to several causes. Specifically, vascular injury is thought to be involved in the pathogenesis of fibrosis. The effects of the antifibrotic drug pirfenidone on angiogenesis have not been fully elucidated. This study aimed to investigate the effects of pirfenidone in human lung fibroblast–endothelial cell co-culture network formation and to analyze the underlying molecular mechanisms. Human lung fibroblasts were co-cultured with human umbilical vein endothelial cells to establish a co-culture network cell sheet. The influence of pirfenidone was evaluated for protective effect on the endothelial network in cell sheets stimulated with transforming growth factor β (TGF-β). Results indicated that TGF-β disrupted the network formation. Pirfenidone and Y27632 (Rho-associated coiled-coil containing protein kinase [Rho-kinase or ROCK] inhibitor) protected against the TGF-β–induced endothelial network disruption. TGF-β activated Rho-kinase signaling in cells composing the co-culture cell sheet, whereas pirfenidone and Y27632 inhibited these effects. In conclusion, TGF-β–induced Rho-kinase activation and disrupted endothelial network formation. Pirfenidone suppressed TGF-β–induced Rho-kinase activity in cell sheets, thereby enabling vascular endothelial cells networks to be preserved in the cell sheets. These findings suggest that pirfenidone has potential vascular network–preserving effect via inhibiting Rho-kinase activity in vascular injury, which is a precursor to pulmonary fibrosis.

## 1. Introduction

Interstitial pneumonia is a collective term for diseases that damage the pulmonary interstitium and are attributed to diverse causes, including idiopathic pulmonary disorders, lung conditions associated with allergic reactions (e.g., hypersensitivity pneumonitis), medications, connective tissue diseases, and sarcoidosis. Pathologically, varying degrees of changes along a continuum from cellular inflammation to fibrosis are observed in interstitial pneumonia. A pathological diagnosis is based on the degree and characteristic findings of inflammatory and fibrotic changes, which are used in conjunction with clinical findings to establish a definitive diagnosis. Interstitial pneumonia that mainly exhibits fibrosis is called fibrosing interstitial lung disease (FILD); in roughly 20–30% of cases of FILD with non-idiopathic pulmonary fibrosis, the progression of fibrosis leads to respiratory failure [1,2]. Because steroid therapy, the most common anti-inflammatory therapy, has minimal effect in FILD [3,4], antifibrotic drugs such as nintedanib [5] and pirfenidone [6,7] are used with the hope of suppressing the progression of disease, although the prognosis in such cases is poor. The median survival time after a diagnosis of idiopathic pulmonary fibrosis (IPF), which chronically worsens, is 3–5 years [8,9]; FILD follows a course similar to that seen in IPF [10].

Pathological tissue that is characteristic of interstitial pneumonia exhibits persistent injuries in both the alveolar epithelium (e.g., due to smoking, air pollution, micro-aspiration, occupational exposures) and the microvascular endothelium (e.g., due to allergies or connective tissue diseases), with interstitial inflammation and abnormal wound healing being the starting points for the eventual development of fibrosis. Initially, fibroblasts, pericytes, epithelial cells, vascular endothelial cells, and fibrocytes transform into myofibroblasts, leading to the development of fibrosis. Myofibroblasts are responsible for excessive production of the extracellular matrix, resulting in lung tissue remodeling. Consequently, hardening and hypoxia of the tissue occur, leading to increased production of profibrotic cytokines and activation of myofibroblasts, thereby initiating an ongoing loop of progressive fibrosis [11,12,13,14,15,16]. Transforming growth factor β (TGF-β), which is produced by the respiratory epithelium and macrophages in response to injury, is an important profibrotic cytokine that is also involved in the pathogenesis of pulmonary fibrosis [17]. Stimulation by TGF-β induces both injury to epithelial cells in the airway and their differentiation into myofibroblasts; TGF-β also causes epithelial mesenchymal conversion and deposition of the extracellular matrix in tissue, leading to fibrosis [17,18].

Abnormal tissue repair due to vascular endothelial dysfunction can induce the formation of fibrotic tissue [19,20]. In a mouse model of bleomycin (BLM)-induced interstitial pneumonia, repeated endotracheal administration of BLM was shown to activate pulmonary capillary endothelial cells and perivascular macrophages, which induced fibrosis by inhibiting alveolar repair [21]. The likely underlying mechanism involves promotion of fibrosis in pulmonary capillary endothelial cells via enhanced Notch signaling by vascular endothelial growth factor receptor 1–expressing perivascular macrophages [21].

The literature contains limited reports describing how antifibrotic agents used in clinical practice affect the vascular network in interstitial pneumonia. Specifically, pirfenidone has been reported to reduce vascular endothelial network formation in vascular endothelial cells, and the inhibitory effect of pirfenidone on tube formation has been studied [22,23]. As these reports were based on studies of pirfenidone in vascular endothelial cells alone, further investigation is necessary to evaluate the real-life pharmacological effects of this agent on the vascular network in interstitial pneumonia. Nintedanib, an antifibrotic drug similar to pirfenidone, has been reported to improve structural abnormalities seen in blood vessels in BLM-induced interstitial pneumonia in a mouse model; it has been proposed that such vascular structural abnormalities may be involved in fibrosis [24]. The in vivo study could not exclude the possibility that the observed findings resulted from multiple signaling pathways; therefore, a simplified experimental system is needed to more fully evaluate these observations.

In a previous study, we co-cultured vascular endothelial cells and fibroblasts and subsequently constructed a vascular endothelial cell network under culture conditions [25]. As vascular endothelial dysfunction, fibroblasts, and TGF-β stimulation are considered to be potentially involved in the pathogenesis of pulmonary fibrosis [17,18,19,20], this co-culture network cell sheet has the potential to provide a simpler model for evaluating the interstitial pathology of the lungs. This cell sheet may thus be useful as an experimental method for quantitatively evaluating such pathologic factors, including the interactions among these various types of cells.

The relationships between the pathology of interstitial pneumonia, vascular dysfunction, and the protective effect of pirfenidone on vascular dysfunction have not been fully investigated. We propose that the use of this cell sheet will help to elucidate this underlying pathology and to clarify new modes of action for pirfenidone. This study aimed to examine effects on the vascular endothelial network under stimulation by TGF-β—a cytokine with an important role in the development of pulmonary fibrosis—as well as the effects and mechanism of action of pirfenidone in cell sheets created from human fibroblasts co-cultured with vascular endothelial cells.

## 2. Materials and Methods

### 2.1. Cell Culture

Human umbilical vein endothelial cells (HUVEC) and human fetal lung fibroblast 1 (HFL1) cells were obtained from the American Type Culture Collection. HUVEC were cultured with a VascuLife^®^ VEGF Endothelial Medium Complete Kit (Lifeline Cell Technology; Frederick, MD, USA), and HFL1 cells were cultured with Ham’s F-12K (Kaighn’s) Medium (Thermo Fisher Scientific Inc., Waltham, MA, USA) with 10% fetal bovine serum albumin. Collagen Type 1 coated plates (Iwaki, Holliston, MA, USA) were used for HUVEC culture. For HFL1 cells, standard culture flasks were used.

### 2.2. Co-Culture Endothelial Network Formation

A cell sheet using HFL1 cells and HUVEC was established using previously described methods [25]. HFL1 cells were seeded in noncoated, 12-well, multiwell plates with F-12K medium at 6.0 × 10^4^ cells/well. On day 2, HUVEC were seeded onto the HFL1 at 4.0 × 10^4^ cells/well with VEGF Comp Kit^®^. The VEGF Comp Kit^®^ medium was changed on day 3. The serum-free medium was replaced on day 6. Recombinant Human TGF-beta 1 Protein (R&D Systems, Inc., Minneapolis, MN, USA) was added on day 7. Pirfenidone (Asahi Kasei Pharma Corporation, Tokyo, Japan) and Y27632 (Rho-associated protein kinase [Rho-kinase or ROCK] inhibitor; FUJIFILM Wako Pure Chemical Corporation, Tokyo, Japan) were added simultaneously. Because pirfenidone was reconstituted in dimethyl sulfoxide, the same amount of dimethyl sulfoxide was added in vehicle control groups. The degree of preservation of network formation was assessed and subsequent cellular analysis was performed on day 8.

### 2.3. Flow Cytometry Analysis

Cells composing the cell sheet were dispersed to single cells and stained with anti-platelet endothelial cell adhesion molecule-1 (PECAM-1)–fluorescein isothiocyanate (FITC) (eBioscience, Inc., San Diego, CA, USA) and anti-human CD144 (VE-cadherin)–allophycocyanin (APC) (eBioscience, Inc.) and then analyzed by fluorescence-activated cell sorter (FACS; FACSCalibur Flow Cytometer; Becton, Dickinson and Company, Franklin Lakes, NJ, USA). The cell sheet was detached using Accumax^®^ (Innovative Cell Technologies, Inc., San Diego, CA, USA), and the reaction was stopped with 10% fetal bovine serum (RPMI-1640 Medium; Sigma-Aldrich, St. Louis, MO, USA). The cells were then resuspended in FACS buffer. PECAM-1–FITC, CD144 (VE-cadherin)–APC, IgG isotype control–FITC, and IgG-APC were incubated with cells for 45 min at room temperature in the dark. Percentages of PECAM-1–FITC and VE-cadherin–APC double positive cells (endothelial cells) among all cells in the cell sheet were measured after 24 h stimulation with TGF-β (1.0 ng/mL) with or without pirfenidone (0.1–10.0 mM). Similarly, PECAM-1–FITC positive cells percentages or VE-cadherin–APC positive cells percentages were measured. Each experiment was independently performed 6 times.

### 2.4. Immunostaining

The immunostaining procedure used in this study has been described in a previous report [25]. Culture plates were fixed with 4% paraformaldehyde for 15 min and rinsed with phosphate-buffered saline (PBS). Cells were permeabilized by adding chilled methanol at −20 °C for 10 min, followed by washing 3 times with PBS and incubating with 1% bovine serum albumin for 1 h. After being washed 3 more times with PBS, the cells were incubated with the primary antibody at the recommended concentration according to the manufacturer’s instructions. Anti-human CD31/PECAM-1 mouse antibody (R&D Systems, Inc.) was used as the primary antibody for HUVEC, and anti–alpha smooth muscle actin (αSMA) rabbit antibody (Abcam, Cambridge, UK) was used for HFL1 cells. After incubation, cells were washed 3 times with PBS and then incubated for 1 h with the secondary antibody (1:50), which was goat anti-rabbit IgG Alexa Fluor^™^ 555 (Invitrogen, Waltham, MA, USA) or goat anti-mouse IgG Alexa Fluor^™^ 488 (Invitrogen).

### 2.5. Evaluation of Endothelial Network

Immunostained endothelial network formation in cell sheets was evaluated with all-in-one fluorescence microscope BZ-X710 (KEYENCE, Osaka, Japan) and Analysis Application BZ-H3A and BZ- H3M (KEYENCE). Cell sheets were immunostained with PECAM-1 and αSMA and documented with low-power field images (approximately 3600 μm × 2700 μm) with BZ-X710 and PlanApo_λ 4× (magnification) 0.20/20.00 mm. Green fluorescence (Alexa Fluor 488, PECAM-1) was detected using a BZX GFP filter OP-87763 (KEYENCE); red fluorescence (Alexa Fluor 555, αSMA) was detected using a BZX Texas Red filter OP-87765 (KEYENCE). Branching number and network length were evaluated using the BZ-X analyzer measurement program BZ-H3A and BZ-H3M. The total network length (µm) in the area (µm^2^) within the images was measured, and the network length per unit area (μm/μm^2^) was calculated. The total number of branches in the area (µm^2^) within the images was calculated (branching number/µm^2^). The endothelial network length and branching number indicate the length of the cell boundary and the complexity of the network structure, respectively. Mean ± standard error of the mean (SEM) values were obtained from 4 independent experimental systems.

### 2.6. Rho-Kinase Activity Assay

Rho-kinase activity was evaluated by ROCK Activity Immunoblot Kit (Cell Biolabs, Inc., San Diego, CA, USA). Cell sheets in a 12-well noncoated plate were stimulated with or without TGF-β (1.0 ng/mL) and pirfenidone (10.0 mM) or Y27632 (10.0 μM) for 24 h. Cell sheets were then lysed with 100 μL of cold cell lysis buffer (Cell Signaling Technology, Danvers, MA, USA) with cOmplete^™^ Protease Inhibitor Cocktail (Roche, Basel, Switzerland). The next steps in the procedure were implemented according to the kit protocol: Using 25 μL of lysate, kinase reaction was initiated by adding 50 μL of 1 × kinase/ATP/substrate solution (myosin phosphatase target subunit 1 [MYPT1]). The tubes were incubated at 30 °C for 60 min with gentle agitation. The kinase reaction was stopped by adding 25 μL of 4× reducing SDS-PAGE sample buffer (Thermo Fisher Scientific). Each sample was boiled for 5 min and then centrifuged. Electrophoresis and transfer were then performed. For immunoblotting and detection, anti-phospho-MYPT1 (Thr696) antibody (Cell Biolabs, Inc.), freshly diluted to 1:1000 in 5% nonfat dry milk/tris buffered saline with Tween 20 (TBST), was used; this was incubated for 2 h at room temperature with constant agitation. The secondary antibody, goat anti-rabbit IgG, horseradish peroxidase–conjugated, was diluted to 1:1000 in 5% nonfat dry milk/TBST and incubated for 1 h at room temperature with constant agitation. Similarly, rabbit anti–glyceraldehyde-3-phosphate dehydrogenase (GAPDH) antibody (Novus Biologicals, LLC, Minneapolis, MN, USA) was used to confirm the amount of protein. The reactants, phosphorylated MYPT1, were analyzed using the image processing program Image J (version 1.53t, US National Institutes of Health), and intensity of membrane were used for results. Mean ± SEM values were obtained from 3 independent experimental systems.

### 2.7. Collagen Assay

Sircol^™^ Soluble Collagen assay kit (Biocolor Ltd., Carrickfergus, UK) was used. Collagen content in co-culture was measured after stimulation with TGF-β (5.0 ng/mL) for 24 h and pirfenidone (10.0 mM) or Y27632 (10.0 μM) in a 12-well plate. For collagen purification, 0.5 M of acetic acid with 0.1 mg/mL of pepsin was added to plates and scraped cell sheets. These samples were incubated for 24 h at 4 °C. Protein concentration was evaluated with the Pierce BCA Protein Assay (Thermo Fisher Scientific). In total, 100 μg of protein solutions (≤1000 μL with distilled water), including 100 μL acid neutralizing reagent (kit contents), was used. A cold isolation and concentration reagent (200 μL/tube, kit contents) was then added, and the contents were incubated overnight at 4 °C. The tubes were centrifuged at 12,000 rpm for 10 min. The supernatant was removed, retaining 100 µL. Then 1 mL of Sircol^™^ Dye Reagent (kit contents) was added to each tube, and the tubes were placed in a gentle mechanical shaker for 30 min. The tubes were centrifuged at 12,000 rpm for 10 min, and the supernatant was removed. Next, 750 μL ice-cold acid-salt wash reagent (kit contents) was added; contents were centrifuged at 12,000 rpm for 10 min. The supernatant was removed, and 250 μL alkali reagent (kit contents) was added. Each sample was transferred to an individual well in a 96-microwell plate. The microplate reader was set to 555 nm, and absorbance of the reagent blanks, standards, and test samples was measured against water. Mean ± SEM values were obtained from 3 independent experimental systems.

### 2.8. Apoptosis Evaluation

Apoptosis of cells was evaluated with the Annexin V-FITC Apoptosis Detection Kit (Abcam). Cell sheets were incubated overnight with TGF-β (1.0 ng/mL), pirfenidone (10.0 mM), and Y27632 (10.0 µM). Cell sheets were detached using 0.25% trypsin at 37 °C for 3 min, and the reaction was stopped with 10% fetal bovine serum (RPMI-1640 Medium, Sigma-Aldrich). Cells were dispersed from cell sheets and then resuspended in 500 μL of 1× binding buffer (kit contents). Then, 5 μL each of Annexin V- fluorescein isothiocyanate (FITC) and propidium iodide (PI)- phycoerythrin (PE) (50 μg/mL, optional) was added; contents were incubated at room temperature for 5 min in the dark. Flow cytometry was then used to measure Annexin V+/PI− cells as an apoptosis cell. Mean ± SEM values were obtained from 3 independent experimental systems.

### 2.9. Statistical Analysis

Each experiment was performed independently at least 3 times. Data were presented as mean values with the corresponding SEMs. Student’s *t* test was used, and statistical significance was defined as *p* < 0.05. Data analysis was performed using Microsoft Excel (version 1.75.2, Microsoft Corporation, 2022).

## 3. Results

### 3.1. Co-Culture Endothelial Network Formation

**Figure 1:**
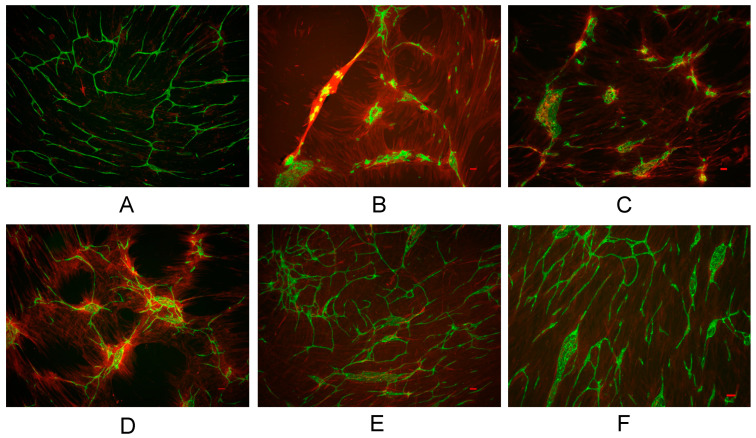
Morphology of vascular endothelial network in co-culture models. Cells were stimulated for 24 h with TGF-β (1 ng/mL), pirfenidone (0.1–10.0 mM), and Y27632 (10 µM). Cells were stained with PECAM-1 (green), αSMA (red). (**A**) Vehicle. (**B**) TGF-β stimulation. (**C**) TGF-β + pirfenidone 0.1 mM. (**D**) TGF-β + pirfenidone 1.0 mM. (**E**) TGF-β + pirfenidone 10.0 mM. (**F**) TGF-β + Y27632 10.0 µM. Scale bar = 100 µm. αSMA, alpha smooth muscle actin; PECAM-1, platelet endothelial cell adhesion molecule-1; TGF-β, transforming growth factor β; Y27632, Rho-kinase inhibitor.

An endothelial network formation model was established by co-culturing HFL1 cells and HUVEC. Cell sheets were stained with PECAM-1 (green) and αSMA (red). TGF-β (1.0 ng/mL) inhibited endothelial network formation, whereas pirfenidone (0.1–10.0 mM) protected the network in a concentration-dependent manner. Y27632 (10.0 µM) also preserved the network, similar to the effect of pirfenidone (Figure 1). Stimulation with TGF-β resulted in endothelial aggregates in cell sheets. Moreover, low concentrations of pirfenidone resulted in poor branching of the endothelial network. TGF-β enhanced αSMA expression, which was reduced by pirfenidone treatment (Figure 1).

### 3.2. Evaluation of Endothelial Network Length and Branch Number in the Co-Culture Model

**Figure 2:**
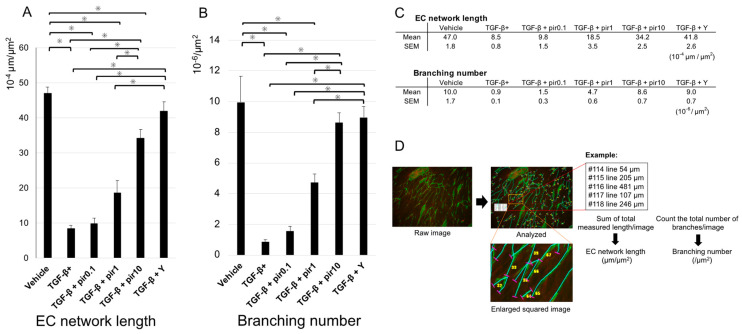
Quantification of vascular endothelial networks in the co-culture model. (**A**) the endothelial cell network length and (**B**) the number of branches per area were measured in the co-culture network formation model. (**C**) Measurements data of network length and Branching number. (**D**) Example of the measurement scheme. Images were taken at low magnification, and the total length of the network and number of branches were measured by an analytical program. The total network length (µm) in the area (µm^2^) in the images taken was measured, and the network length per unit area (μm/μm^2^) was calculated. The total number of branches in the area (µm^2^) in the images taken was calculated (number of branches/µm^2^). TGF-β reduced network length and number of branches; pirfenidone improved these parameters in a concentration-dependent manner, and Y27632 also had a beneficial effect. Data shown are mean values ± SEM (*n* = 4). * *p* < 0.05. EC, endothelial cell; pir0.1, pirfenidone 0.1 mM; pir1, pirfenidone 1.0 mM; pir10, pirfenidone 10.0 mM; TGF-β, transforming growth factor β; TGF-β+, transforming growth factor β stimulation; Y, Y27632 (Rho-kinase inhibitor) 10 µM.

Measurements data of network length/10^−4^ μm^2^ were presented in Figure 2A,C. The network length was reduced by TGF-β but preserved by pirfenidone in a concentration-dependent manner (*p* < 0.05). Y27632 also improved the network length (*p* < 0.05) (Figure 2A).

Measurements data of the numbers of branches/10^−6^ μm^2^ were presented in Figure 2B,C. Branching numbers were reduced by TGF-β treatment and preserved in a concentration-dependent manner by treatment with pirfenidone (*p* < 0.05). Y27632 also improved the number of branches (*p* < 0.05) (Figure 2B). An example of the measurement scheme was described in Figure 2D.

### 3.3. Assessment of Cell Proportions in Cell Sheet of Co-Culture Model

**Figure 3:**
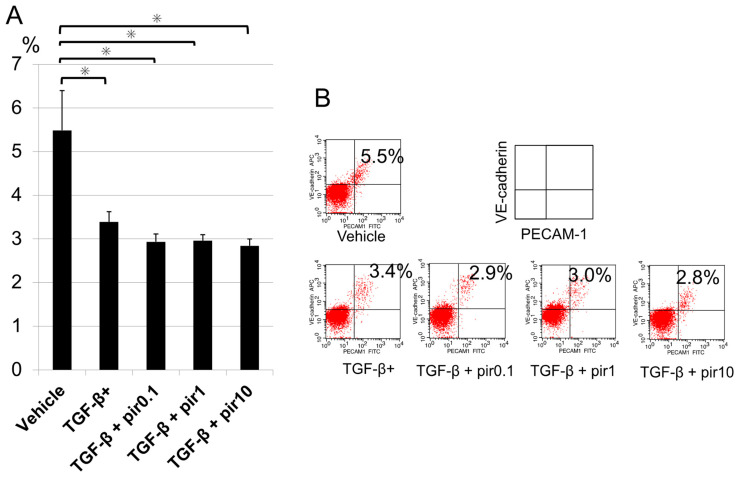
Evaluation of vascular endothelial cell percentage during TGF-β stimulation and pirfenidone administration. Percentages of PECAM-1-FITC and VE-cadherin–APC positive cells (endothelial cells) to total cells in cell sheets were measured after stimulation for 24 h with TGF-β (1.0 ng/mL) and pirfenidone (0.1–10.0 mM). (**A**) Graphical representation. (**B**) Flow cytometry results. Data shown are mean values ± SEM (*n* = 6). * *p* < 0.05. APC, allophycocyanin; VE, vascular endothelial; FITC, fluorescein isothiocyanate; PECAM-1, platelet endothelial cell adhesion molecule-1; pir0.1, pirfenidone 0.1 mM; pir1, pirfenidone 1.0 mM; pir10, pirfenidone 10.0 mM; TGF-β, transforming growth factor β; TGF-β+, transforming growth factor β stimulation.

Percentages of PECAM-1-FITC and VE–Cadherin-APC double positive cells (endothelial cells) to total cells in the cell sheets were measured after 24 h stimulation with or without TGF-β (1.0 ng/mL) and pirfenidone (0.1–10.0 mM). These results were as follows: vehicle, 5.5 ± 0.9%; TGF-β stimulation, 3.4 ± 0.2%; TGF-β + pirfenidone 0.1 mM, 2.9 ± 0.2%; TGF-β + pirfenidone 1.0 mM, 3.0 ± 0.1%; TGF-β + pirfenidone 10.0 mM, 2.8 ± 0.2%. TGF-β reduced the proportion of vascular endothelial cells (*p* < 0.05) (Figure 3). In addition, pirfenidone administration decreased the percentages of VE-cadherin or PECAM-1 in TGF-β + pirfenidone 10.0 mM versus vehicle, respectively (*p* < 0.05) (Figure S2).

### 3.4. Rho-Kinase Activity in Co-Culture Model

**Figure 4:**
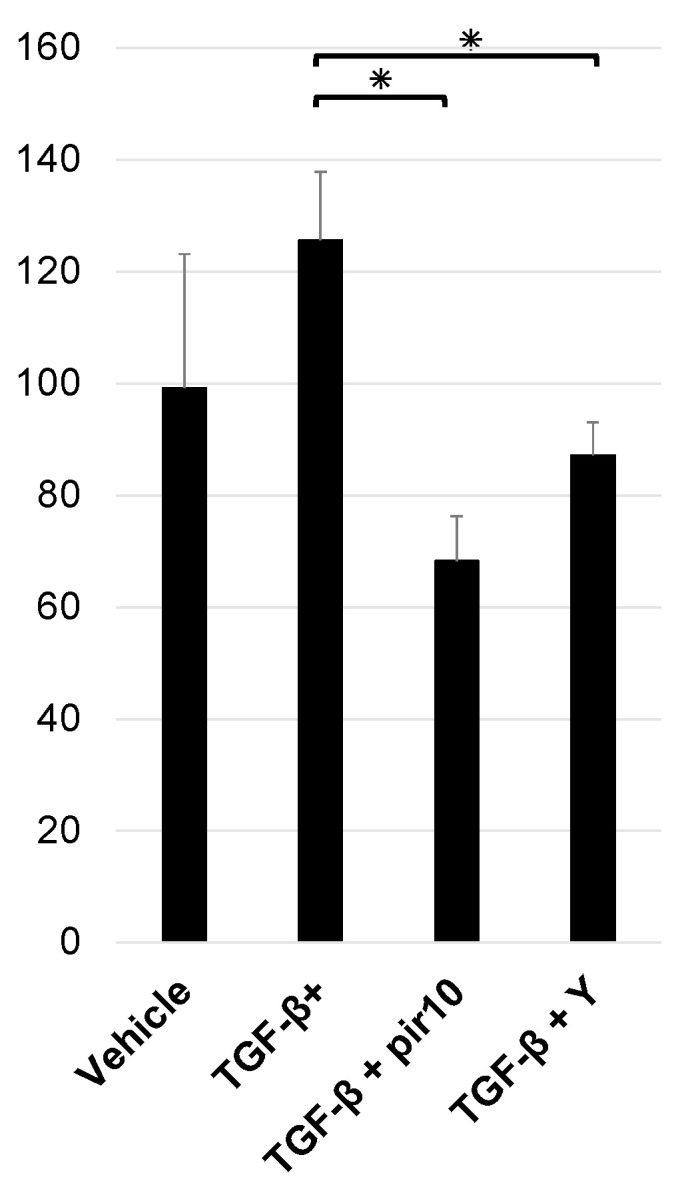
Rho-kinase activity in the cell sheet. Rho-kinase activity was assessed after stimulation with TGF-β (1.0 ng/mL) for 24 h. Rho-kinase activity was higher with TGF-β and improved with pirfenidone 10 mM and Y27632 (Rho-kinase inhibitor) 10 μM. Data shown are mean values ± SEM (*n* = 3). * *p* < 0.05. pir10, pirfenidone 10.0 mM; TGF-β, transforming growth factor β; TGF-β+, transforming growth factor β stimulation; Y, Y27632 (Rho-kinase inhibitor) 10.0 µM.

Measurements of Rho-kinase activity after stimulation with TGF-β (1.0 ng/mL), pirfenidone (10.0 mM), and Y27632 (10.0 μM) were as follows: vehicle, 99.2 ± 24.0; TGF-β stimulation, 125.6 ± 12.2; TGF-β + pirfenidone 10 mM, 68.3 ± 8.0; TGF-β + Y27632 10 μM, 87.1 ± 6.0. Rho-kinase activity was higher with TGF-β and significantly decreased with pirfenidone or Y27632 (*p* < 0.05) (Figure 4).

### 3.5. Collagen Production in the Co-Culture Model

**Figure 5:**
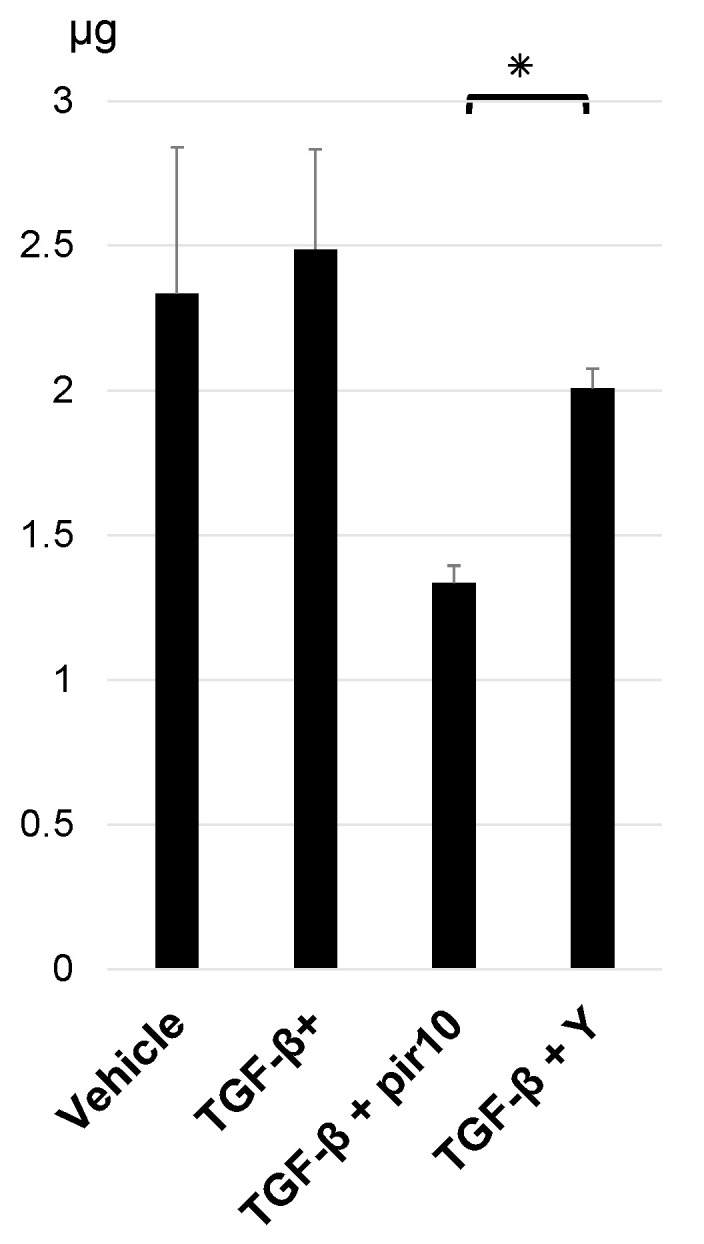
Collagen production in cell sheets. Collagen content in co-culture cell sheets was measured after stimulation for 24 h with TGF-β (5.0 ng/mL), pirfenidone 10.0 mM, or Y27632 (Rho-kinase inhibitor) 10.0 μM. Data shown are mean values ± SEM (*n* = 3). * *p* < 0.05. pir10, pirfenidone 10.0 mM; TGF-β, transforming growth factor β; TGF-β +, transforming growth factor β stimulation; Y, Y27632 (Rho-kinase inhibitor) 10.0 µM.

Measurements of collagen production in cell sheets after 24 h of stimulation with TGF-β (5.0 ng/mL), pirfenidone (10.0 mM), or Y27632 (10.0 μM) were as follows: vehicle, 2.3 ± 0.5 μg; TGF-β stimulation, 2.5 ± 0.3 μg; TGF-β + pirfenidone 10 mM, 1.3 ± 0.1 μg; TGF-β + Y27632 10 μM, 2.0 ± 0.1 μg. Collagen content was higher, but not significantly, in TGF-β-treated cell sheets and lower in cell sheets treated with pirfenidone or Y-27632 (Figure 5).

### 3.6. Measurement of Apoptosis in the Co-Culture Model

Co-cultured cells were stimulated overnight with TGF-β (1.0 ng/mL), pirfenidone (10.0 mM), and Y27632 (10.0 µM). Annexin V positive (+)/PI negative (−) cells were measured as an apoptosis cell by flow cytometry. Measured values: vehicle, 6.7 ± 0.7%; TGF-β+, 7.9 ± 0.4%; TGF-β + pir10, 6.2 ± 1.4%; TGF-β + Y, 6.7 ± 0.3%. No significant differences were observed in this study (Figure S1).

## 4. Discussion

This study revealed that pirfenidone, a therapeutic agent used in the treatment of IPF, acted protectively against the inhibitory effect of TGF-β on vascular endothelial network formation, which plays an important role in promoting the development of fibrotic tissue. The protective effect is considered to be mediated by Rho-kinase, and the disruption of the vascular endothelial network formation by the Rho cascade could lead to fibrosis. However, it should be noted that disruption of the endothelial network is not the only etiological factor of developing fibrosis. Other possibilities, such as endothelial dysregulation, loss of connectivity and endothelial-mesenchymal transition leading to blood leakage and immune cell infiltration into the injury site, also contribute to pulmonary fibrosis [26].

Pirfenidone is the first drug to show prolonged survival and reduced decline in respiratory function in IPF [27]. The pharmacological effects of pirfenidone include suppressed production of TGF-β from alveolar epithelial cells and vascular endothelial cells [28,29,30], inhibited TGF-β1/SMAD3 signaling-mediated differentiation of fibroblasts into myofibroblasts as well as reduced fibroblast proliferation [31], and reduced production of extracellular matrix proteins (tenascin-c and fibronectin) in response to TGF-β stimulation in fibroblasts [32]. Additionally, pirfenidone is involved in inhibiting fibrosis via suppressing the production of platelet-derived growth factors (i.e., growth factors of fibroblasts derived from alveolar macrophages) [33] as well as basic fibroblast growth factor and TGF-β in lung tissue [34]. Macrophages present in the respiratory tract are considered to be important in pulmonary fibrosis, as they produce various cytokines and chemokines and induce wound healing and immune responses [35]. Pirfenidone has also been reported to have an antifibrotic effect [18] via inhibiting interleukin-1, tumor necrosis factor α, TGF-β, and platelet-derived growth factor—all of which are inflammatory cytokines produced by alveolar macrophages [33,36]—as well as chemokines such as monocyte chemoattractant protein-1 [37].

To date, the influence of pirfenidone on the vascular endothelium has not been sufficiently examined; however, pirfenidone has been suggested to act on the vascular endothelium in a biphasic fashion [22,23]. Pirfenidone has been shown to promote angiogenesis at low concentrations and to inhibit neoangiogenesis at high concentrations [23]. High concentrations of pirfenidone significantly inhibit the expression of key migration cytokines, matrix metalloproteinases (MMPs) MMP-2 and MMP-9; by contrast, low concentrations of pirfenidone significantly promote expression of these substances [23]. High concentrations of pirfenidone have also been reported in previous study to promote apoptosis [23]. In that study, high concentrations of pirfenidone were defined as 10–100 μM, which differs from the concentrations used in our experimental system. As our study was conducted under co-culturing conditions, it is possible that the optimal concentration of pirfenidone differed on the basis of these conditions. The cytotoxicity of pirfenidone in the previous data showed that inhibition of proliferation and decreased motility of pirfenidone-treated fibroblasts were observed, but cell death was not, which was different from a report by Gan D et al. [23,38]. In our study using a co-culture model, no enhancement of apoptosis was observed with pirfenidone at 10.0 mM. TGF-β stimulation reduced the percentage of vascular endothelial cells among all cells composing the cell sheet; in each experimental group (vehicle, TGF-β stimulation, and TGF-β + pirfenidone 0.1–10.0 mM), however, no decrease in the total number of cells was observed. These findings suggest that the mechanism of action of the inhibitory effect of pirfenidone on endothelial cell network disruption due to TGF-β may involve maintaining the morphology of the vascular endothelial cell network. Pirfenidone administration protected network formation but decreased the percentage of VE-cadherin, a marker of endothelial barrier dysfunction and PECAM-1 [39]. It is possible that pirfenidone affects the expression of adhesion molecules in the vascular endothelium. It has been reported that endothelial barrier dysfunction is key to macrophage recruitment into the lung [40], and further studies are needed on the effects of expression of adhesion factors.

Several papers have reported that Rho-kinase inhibitors suppress pulmonary fibrosis by suppressing blood vessel leakage or immune cell infiltration into the injury site. [41,42]. Clinically, Rho-kinase activity is increased in lung tissue from patients with IPF and is thought to lead to Notch1-mediated lung fibrosis [43,44]. This increased Rho-kinase imply its importance as a therapeutic target. In vascular endothelial cells, Rho-kinase is involved in cell proliferation, non-muscle myosin-mediated cell migration, morphological changes, and cell permeability [45,46,47]. As a mechanism for inducing vascular permeability, stimulation of vascular endothelial cells by TGF-β has been reported to promote filamentous actin via increased RhoA/Rho-kinase activity [46]. Increased vascular permeability also is a feature of tissue changes that occurs in acute lung injury [46]. In our study, we also observed increased Rho-kinase activity due to TGF-β, suggesting that a similar phenomenon may occur in vascular endothelial cells. In myofibroblasts, Rho-kinase has been reported to be involved in myofibroblast transdifferentiation and the promotion of fibrosis [48]. Fibroblasts transform into myofibroblasts when stimulated by TGF-β, resulting in increased collagen production. Similarly, in the present study, increased collagen production due to TGF-β in the cell sheet was observed; this increase was suppressed by the Rho-kinase inhibitor. Based on these findings and given the suppression of collagen production by the Rho-kinase inhibitor, it is possible that the cascade of promoting fibrosis via activation of Rho-kinase may have been inhibited in fibroblasts composing the cell sheet.

**Figure 6:**
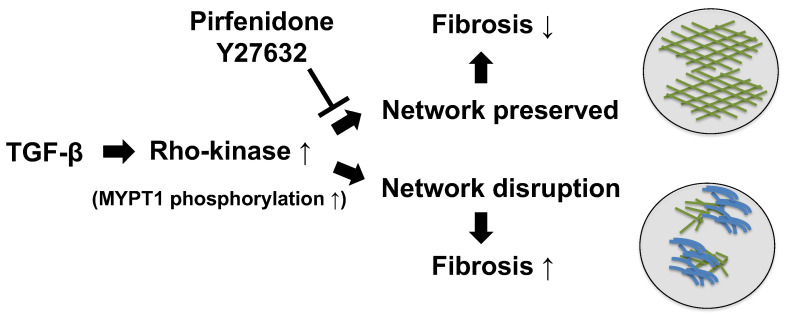
Hypothetical overview of the effects of TGF-β on the cell sheet and the protective effect of pirfenidone and Y27632 against TGF-β. TGF-β stimulation activates Rho-kinase, which disrupts the endothelial network and promotes pulmonary fibrosis. Pirfenidone or Y27632 preserves the endothelial network via inhibiting Rho-kinase activity, resulting in suppressed pulmonary fibrosis. MYPT1, myosin phosphatase targeting subunit 1; TGF-β, transforming growth factor β; Y27632, Rho-kinase inhibitor.

It is likely that the inhibitory effect of TGF-β on the formation of vascular endothelial network and the protective effect associated with Rho-kinase inhibition observed in this study affected both fibroblasts and vascular endothelial cells. Given that TGF-β stimulation was independent of the percentage of vascular endothelial cells in the cell sheet, it is possible that the inhibition of actin stress fiber formation via Rho-kinase may have been involved in vascular endothelial cells [49,50]. It was also confirmed that fibroblasts subjected to TGF-β stimulation undergo changes in morphology, with gaps observed between the cells, suggesting that fibroblasts may also be involved in destruction of the vascular endothelial network. In other words, HFL1 cells may undergo morphological changes due to TGF-β stimulation, and the vascular endothelial cell network may no longer be retained because of morphological changes in fibroblasts, which serve as scaffolds for the vascular endothelial cell network. The present study revealed a new effect of pirfenidone, as no prior study has reported the role of pirfenidone in maintaining the vascular endothelium network via inhibiting Rho-kinase (Figure 6).

Basic research studies regarding the pulmonary vascular network as well as studies of clinical specimens have been reported in the literature. In surgical lung biopsies in patients with IPF, the density of alveolar capillary endothelial cells has been reported to be increased in areas with minimal fibrosis and decreased in areas with more extensive fibrosis [51]. Additionally, in areas with high alveolar capillary endothelial cell densities, the vascular endothelial growth factor is abundantly expressed in capillary endothelial cells [51]. These results suggest that changes in the vascular network and localized changes in cytokine production in tissue may play a role in the pathogenesis of interstitial pneumonia [19]. It is possible that the disruption of the vascular endothelial network in the cell sheet, which was created with fibroblasts serving as scaffolds, as seen in the present study may partly mimic the disease state that is associated with the effect of TGF-β on the vascular network in patients with interstitial pneumonia.

We did not sort out the mixed cells in the measurement of Rho-kinase, which is a limitation of the present study. As previously mentioned, Rho-kinase can potentially affect both the vascular endothelium and fibroblasts; therefore, future research is needed to evaluate which cells are more strongly affected. Additionally, the present study used HUVEC; however, it would be much more accurate methodologically to use human lung microvascular endothelial cells. We stimulated 5 ng/mL TGF-β in the collagen assay because no difference in the amount of collagen was observed at a 1 ng/mL TGF-β. As the collagen assay is a different study condition from the assessment of network formation, further studies are needed to clarify the relationship between network formation and collagen content. In the present study, we focused on TGF-β-induced network disruption, which was protected by pirfenidone, suggesting the involvement of Rho-kinase in this process. To elucidate this mechanism, further studies are needed on the function of Rho-kinase and how it works (e.g., endothelial-mesenchymal transition and cleavage of junctional molecules in vascular endothelial cells). Moreover, this study did not in detail examine fibrosis signaling, in which Rho-kinase activating signaling is involved.

In the vascular endothelium, lysophosphatidic acid (LPA), a phospholipid, has been reported to activate the Rho-kinase cascade and TGF-β [52,53]. As compared with the BLM-induced interstitial pneumonia model, LPA receptor knockout mice show decreased TGF-β in bronchoalveolar lavage fluid (BALF) and decreased collagen in lung tissue; therefore, it is inferred that LPA may promote TGF-β induction and fibrosis [54]. These reports, together with our data demonstrating the activation of Rho-kinase by TGF-β, suggest that the LPA–TGF-β–Rho-kinase cascade may be involved in fibrosis. Furthermore, in BLM-induced interstitial pneumonia model mice, significantly increased levels of autotaxin, an enzyme that converts lysophosphatidylcholine (LPC) in BALF into LPA, has been observed [55], suggesting also that lipid metabolism may be associated with fibrosis [18]. As LPC has been reported to be important in angiogenesis as well [56], it is also possible that changes in the balance of the lipid composition (LPC, LPA metabolism) may affect vascular network formation. Taken together, the LPC–LPA–TGF-β–Rho signaling- vascular endothelial dysfunction signal transduction may be responsible for the pathogenesis of FILD. Further investigations conducted from the perspective of vascular endothelial network inhibition based on lipid metabolism are needed in the future.

## 5. Conclusions

A vascular endothelial cell network model was created from a co-culture using human vascular endothelial cells and lung-derived fibroblasts. When the co-culture model was stimulated by TGF-β, Rho-kinase was activated, and the network formation was disrupted. Findings suggested that pirfenidone has a potential protective effect against the inhibitory influence of TGF-β on network formation, as occurs via Rho-kinase activation.

## Data Availability

The data presented in this study are available on request from the corresponding author.

## Supplementary Materials

The following supporting information can be downloaded at: https://www.mdpi.com/article/10.3390/biomedicines11082259/s1, Figure S1: Measurement of apoptosis in co-culture model. Figure S2: Evaluation of VE-cadherin or PECAM-1 positive cell percentage during TGF-β stimulation and pirfenidone administration.
