# Supplementary material for: A Protective Effect of Pirfenidone in Lung Fibroblast–Endothelial Cell Network via Inhibition of Rho-Kinase Activity
Source: Biomedicines. 2023 Aug 12;11(8):2259. doi: 10.3390/biomedicines11082259 (PMC10452915; doi:10.3390/biomedicines11082259)
Supplement: Supplementary file 1 [file biomedicines-11-02259-s001.zip › biomedicines-2527906-supplementary.pdf]

Supplemental Figures

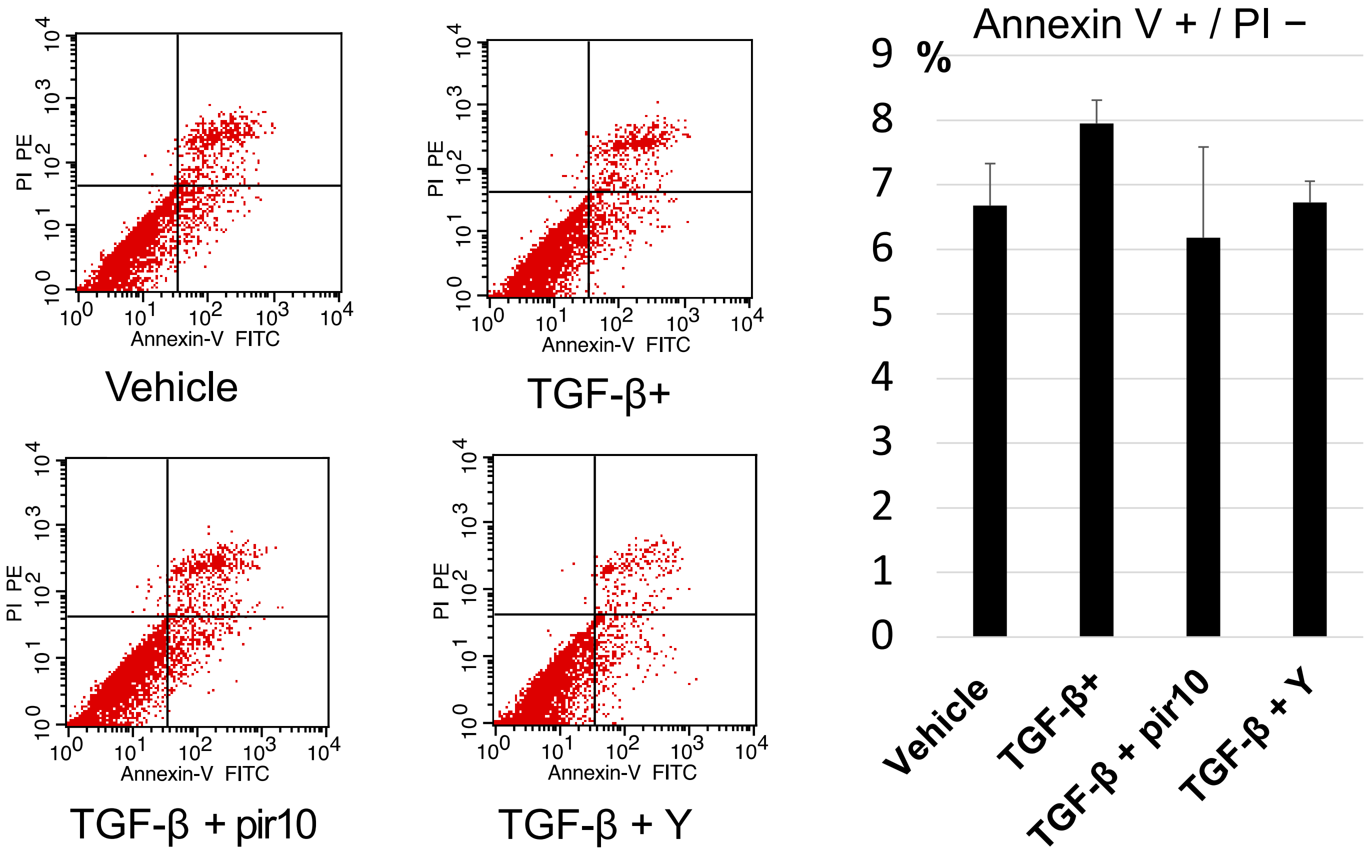

Figure S1. Measurement of apoptosis in co-culture model.

Co-cultured cells were stimulated overnight with TGF-β (1.0 ng/mL), pirfenidone (10.0 mM), and Y27632 (10.0 μM). Annexin V + / PI − cells were measured as an apoptosis cell by flow cytometry. Data shown are mean values ± SEM (n=3).

FITC, fluorescein isothiocyanate; PI, propidium iodide; PE, phycoerythrin; pir10, pirfenidone 10 mM; TGF-β, transforming growth factor β; TGF-β+, transforming growth factor β stimulation; Y, Y27632 (Rho-kinase inhibitor).

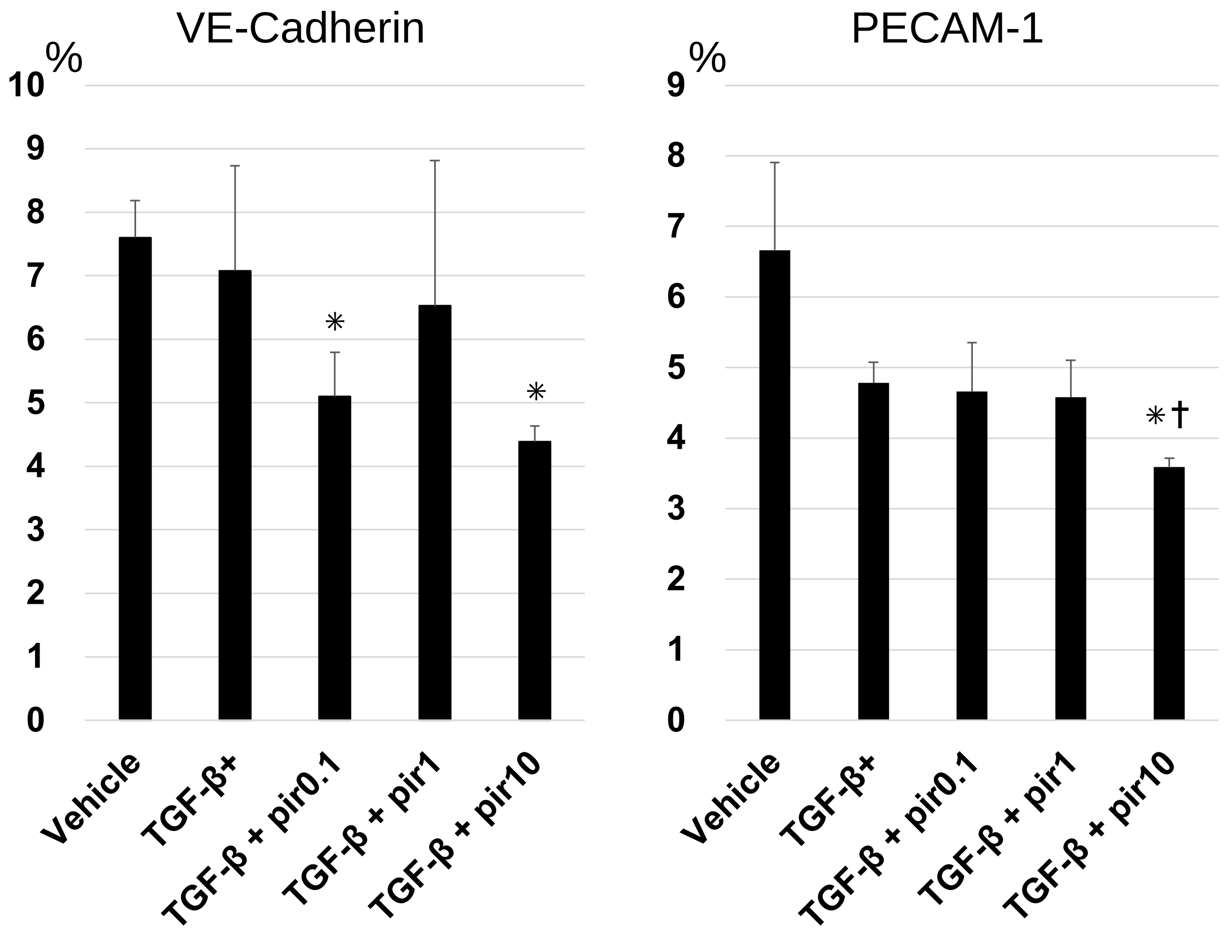

Figure S2. Evaluation of VE-cadherin or PECAM-1 positive cell percentage during TGF-β stimulation and pirfenidone administration.

Percentages of PECAM-1-FITC or VE-cadherin–APC positive cells to total cells in cell sheets were measured after stimulation for 24 hours with TGF-β (1.0 ng/mL) and pirfenidone (0.1-10.0 mM). Data shown are mean values ± SEM (n = 6). *; versus Vehicle ($p < .05$), †; versus TGF-*β* +($p < .05$). APC, allophycocyanin; VE, vascular endothelial; FITC, fluorescein isothiocyanate; PECAM-1, platelet endothelial cell adhesion molecule-1; pir0.1, pirfenidone 0.1 mM; pir1, pirfenidone 1.0 mM; pir10, pirfenidone 10.0 mM; TGF-β, transforming growth factor β; TGF-β+, transforming growth factor β stimulation.
